# The Diel Activity Pattern of *Haemaphysalis longicornis* and Its Relationship with Climatic Factors

**DOI:** 10.3390/insects15080568

**Published:** 2024-07-26

**Authors:** Byung-Eon Noh, Gi-hun Kim, Hak Seon Lee, Hyunwoo Kim, Hee-Il Lee

**Affiliations:** Division of Vectors and Parasitic Diseases, Korea Disease Control and Prevention Agency, 187 Osongsaengmyeong 2-ro, Osong-eup, Heungdeok-gu, Cheongju 28159, Republic of Korea; nbudia@korea.kr (B.-E.N.); kgh7895@korea.kr (G.-h.K.); hslee8510@korea.kr (H.S.L.); hyunwookim@korea.kr (H.K.)

**Keywords:** diel activity, *Haemaphysalis longicornis*, tick surveillance

## Abstract

**Simple Summary:**

Many tick species pose a threat to public health by transmitting diseases to humans and animals. *Haemaphysalis longicornis* (The Asian longhorned tick) is known to be the main vector that transmits severe fever with thrombocytopenia syndrome virus. In the United States, it was first identified in New Jersey during 2017 on domestic sheep. Even though *H. longicornis* is an important vector, research on its major activity period is lacking. Tick activity patterns may vary depending on climate factors, the host, and region. This study aimed to survey the patterns of diel activity in *H. longicornis* and the potential effects of climatic factors on tick activity. The highest activity occurred at 10:00 to 14:00. Avoiding the times of highest activity is considered the best way to reduce contact with ticks.

**Abstract:**

*Haemaphysalis longicornis* is one of the most medically important carriers of various pathogens. Although *H. longicornis* is an important vector, only basic ecological and biological information has been obtained, primarily focusing on its abundance and distribution. This study determined the most active time and meteorological conditions for the diel activity of *H. longicornis*. The diel activity pattern of *H. longicornis* was the highest between 10:00 and 14:00, and the lowest between 22:00 and 02:00. The major activity temperature of *H. longicornis* was between 25 °C and 40 °C, with the highest activity at 35 °C. The relative humidity was between 30% and 70% during the active period. Temperature had the highest correlation with diel activity (R = 0.679), followed by humidity (R = −0.649) and light intensity (R = 0.572). Our results provide basic information for the development of tick-borne disease vector control programs and tick surveillance.

## 1. Introduction

Ticks transmit numerous viral, bacterial, and protozoan pathogens to humans and other animals. As the twenty-first century advances, tick-borne viruses and novel viruses will definitely emerge [1], such as severe fever with thrombocytopenia syndrome (SFTS), Heartland virus, Crimean Congo hemorrhagic fever virus (CCHFV), and Powassan virus. Additionally, Jingmen tick virus [2], Yezo virus [3], Toyo virus [4], and Songlin virus [5] have been identified in China and Japan.

Ticks are increasing in density and expanding their geographical distribution owing to climate change [6,7]. Additionally, global warming causes increased tick activity and changes in tick development, reproduction, and survival rates, as well as changes in the development rates of pathogens that are carried by vectors [7,8]. *Ixodes ricinus* is widely distributed in northwestern Europe, and climate change has expanded its range northward into Norway [9]. The black-legged tick *I. scapularis* has been recorded over an expanded geographic range in the eastern-to-northern parts of the USA over the last half of the century [10]. Additionally, an invasion of the red sheep tick (*Haemaphysalis punctata*) was first identified at Rhode Island, USA in 2010 [11]. After the Asian long-horned tick (*Haemaphysalis longicornis*) was first reported in New Jersey [12], the distribution area has expanded to the southern area of the USA and was recently confirmed in Georgia [13]. Modeling analysis using climate data shows that *H. longicornis* has a broad potential distribution in the United States and Mexico [14]. Currently, there is great concern regarding the potential role of *H. longicornis* as a vector for transmitting pathogens to humans and domestic animals.

*H. longicornis* Neumann is distributed across eastern China, Japan, the Russian Far East, and the Republic of Korea (ROK). It was introduced to Australia over a century ago [15,16,17]. *H. longicornis* was first discovered in the United States in 2017 [12] and has expanded to various regions [18,19,20,21]. Because *H. longicornis* is a three-host Ixodid tick, each life stage (adult, nymph, and larva) must include feeding with blood for development into the next life stage [16]. Furthermore, exotic populations of *H. longicornis* commonly reproduce asexually via parthenogenesis [16,22]. Typically, in the summer, adult females lay 1000 to 4000 eggs in moist soil [23]. Laboratory studies have shown that eggs hatch approximately 25 days after oviposition when stored at 25 °C [24]. Larvae that hatch in the field immediately search tall grass or shrubs for a host [16,25,26]. They can often be found in clusters browsing together at the tips of plants [26]. After blood feeding, the larvae molt into nymphs and typically undergo diapause during the winter [16]. Field surveys demonstrate that *H. longicornis* overwinters mainly at the nymphal stage in the topsoil layer [27]. In late winter or early spring, depending on the temperature and photoperiod, the nymphs become active again and quest for new hosts [16], such as cattle, sheep, deer, or horses [25]. Generally, one generation occurs over the course of a year as ticks undergo a diapause to increase their chances of survival [16,28].

Additionally, *H. longicornis* is one of the most medically and veterinary important vectors that can transmit various pathogens, including *Anaplasma bovis*, *A. phagocytophilum*, *Babesia ovata*, *Bartonella grahamii*, *Ba. henselae*, *Borrelia burgdorferi*, *Bo. miyamotoi*, *Coxiella burnetii*, and *Ehrlichia chaffeensis* [29,30,31,32,33,34]. *H. longicornis* is the main vector of the SFTS virus, which has recently emerged as a lethal pathogen in the ROK, Japan, and China. SFTS was first identified in a patient in China, 2009 [34,35]. In 2013, SFTS cases were first reported in the ROK and Japan, and there were a number of cases in East Asia and other regions, including Thailand, Vietnam, and Myanmar [36,37,38,39,40]. In the ROK, 1895 patients were reported between 2013 and 2023, with a mortality rate of 18.7% [41]. In China, 7721 laboratory-confirmed SFTS cases were reported in 2010–2018, with an overall mortality rate of 10.5% [42]. Between 2010 and 2019, 13,824 SFTS cases were reported in mainland China, with a nationwide average annual fatality rate of 5.2% [43]. In Japan, 174 patients with SFTS were investigated between 2013 and 2021, with a mortality rate of 35% [44]. The highest number of cases was 116 in 2022 [45].

The risk of tick-borne diseases in humans is influenced by many factors; however, the final issue is the questing activity time of unfed ticks. Therefore, studies have been conducted on the diel activity patterns of various species. Questing activities are influenced by climatic and environmental factors. The endogenous rhythm of the ticks and meteorological factors (particularly the temperature, humidity, and insolation) are considered to be the main parameters governing tick diel activity [46,47]. Humidity is one of the most influential factors affecting questing activity and tick survival [48]. Additionally, the activity and behavior of the host may be important [47]. Studies on the activity patterns of ticks have been conducted in several species, and the activity patterns greatly differ for each species. For example, the adult stages of the *Dermacentor reticulatus* peak activity occurs at 18:00–18:25 in spring and 14:00–14:25 in autumn [49]. *Ixodes scapularis* exhibited the highest activity in the early morning, whereas contrast *Amblyomma americanum* nymphs showed peak activity around noon [50]. *I. ricinus* activity peaked between 23:00 and 03:00 [51]. Although *H. longicornis* is an important vector, only basic ecological and biological information has been obtained, primarily focusing on its abundance and distribution.

This study aimed to survey the patterns of diel activity in *H. longicornis* and the potential effects of climatic factors on tick activity. Ultimately, we could identify the major activity times of ticks, provide guidelines to reduce the risk of contact between humans and ticks, and prevent the spread of tick-borne infectious diseases.

## 2. Materials and Methods

### 2.1. Study Site

**Figure 1:**
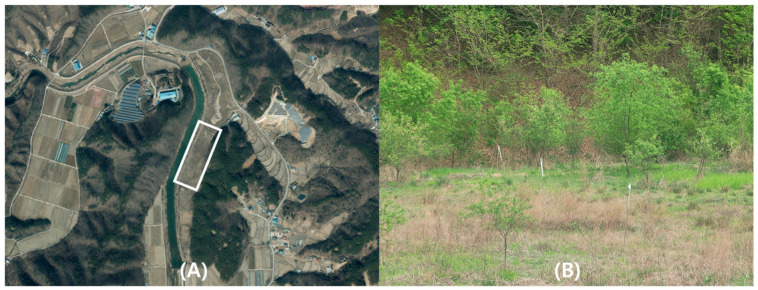
Map of the study site where the traps were installed. (**A**) The location of the site shown broadly and the white box is the actual survey location (image source by Korea Statistical Information Service). (**B**) Panoramic view of the study site.

This study was conducted in Iin-myeon, GongJu-si, Chungcheongnamd-do, Republic of Korea (36°24′45′′ N, 127°04′05′′ E). This area was selected as a suitable environment for hosts because there are resting places and water supply sources around it (Figure 1). Additionally, several ticks were collected from a previous study. The collection site was covered with grass and shrubs, with an area of approximately 0.045 km^2^. A 50 × 70 m experimental section was established at the center.

### 2.2. Sampling Methods and Identification

The ticks were collected using a dry-ice bait trap. The main body of the trap was a white tarpaulin cylinder with an open top (36 cm diameter × 40 cm height). Additionally, a cylindrical ice chest (10 cm diameter × 30 cm height) containing 3 pieces of dry ice (ca, 2.5 kg total) was placed inside the trap to attract ticks [52]. 

**Figure 2:**
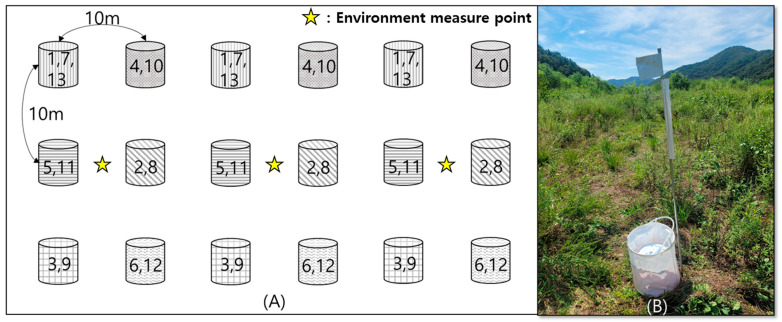
Sampling location method (1: 10:00–12:00, 2: 12:00–14:00, 3: 14:00–16:00, 4: 16:00–18:00, 5: 18:00–20:00, 6: 20:00–22:00, 7: 22:00–24:00, 8: 24:00–02:00, 9: 02:00–04:00, 10: 04:00–06:00, 11: 06:00–08:00, 12: 08:00–10:00, 13: 10:00–12:00) and environmental measure point. (**A**) Dry ice bait trap and stick with reflective tape. (**B**) The ticks collected during the last period were used for identification in the laboratory.

The study was conducted on the third week of every month from April to September at 4 h intervals for 24 h in 2022. The experiment was performed randomly on sunny days. If it rained, we changed the collection period. In August, tick collection could not be conducted owing to rain and wet ground from the rainy season. The attracted ticks (only adults and nymphs) were counted and released 5 m away from the trap to minimize the effects of ticks being removed during collection. The distance between the traps was set to 20 m because the attraction distance of the ticks to dry ice was up to 8 m [17]. To eliminate site bias, the traps were moved to different sites for each time period (Figure 2A). Each trap had a pole with reflective tape to easily find a trap at night (Figure 2B).

For identification, the ticks collected during the last period were brought to the laboratory and identified to the species and life stage levels using a stereomicroscope (Olympus, Tokyo, Japan) and morphological keys [53]. 

### 2.3. Environmental Measurements

The temperature (°C) and relative humidity (RH) (%) were measured at knee height (trap height) near the dry-ice trap using CEM DT-172 (CEM, Shenzhen, China) at three points at the collection site. The light intensity was measured using a CEM DT-8808 (CEM, Shenzhen, China) at the same location. The temperature, humidity, and light intensity were also measured when ticks were counted from the trap.

### 2.4. Statistical Analysis

The collection results were analyzed at 4 h intervals, and the collected ticks at each time period were compared using an analysis of variance (ANOVA). The mean of each group was used in this test. The relationships between the climatic variables were examined using Pearson’s correlation coefficients. We used multiple linear regression to examine the relationships between climatic variables and the number of ticks collected. 

## 3. Result

A total of 412 ticks were collected during the last period, including 53 adults and 376 nymphs. A total of 396 individuals were *H. logicornis* (48 adults and 365 nymphs) and 16 individuals were *H. flava* (five adults and 11 nymphs). From April to July, all ticks were *H. longicornis*. In September, 16 of the 64 ticks collected were *H. flava* and the remaining 48 were *H. longicornis*.

Comparing daytime and nighttime, the *t*-test results confirmed that tick activity patterns were significantly (*p* < 0.01) higher during the daytime in all life stages (adult: t = 3.541, *p* = 0.001, df = 178, nymph: t = 3.712, *p* < 0.001, df = 178, total: t = 4.024, *p* < 0.001, df = 178) (Table 1).

The overall activity pattern was the highest in June, and the lowest activity pattern occurred in April and October. Comparing time periods, the 10:00–14:00 time period had the highest tick activity (average collected: 37.8), followed by 14:00–16:00 (30.9), 16:00–10:00 (26.7), 18:00–22:00 (9.9), 02:00–06:00 (7.7), and 22:00–02:00 (2.8). The average temperature during the study period was 24 °C, the lowest temperature was 5.7 °C in September, and the highest temperature was 42.1 °C in June. The average temperature was over 20 °C, except for April, and the daily temperature range was between 5.7–36.5 °C, with the highest being in April. The average relative humidity at the time of the study was 65.6% and ranged from 11.0 to 100.0%. During the survey period, the highest relative humidity was 100%, except in May and September (Table 2).

**Figure 3:**
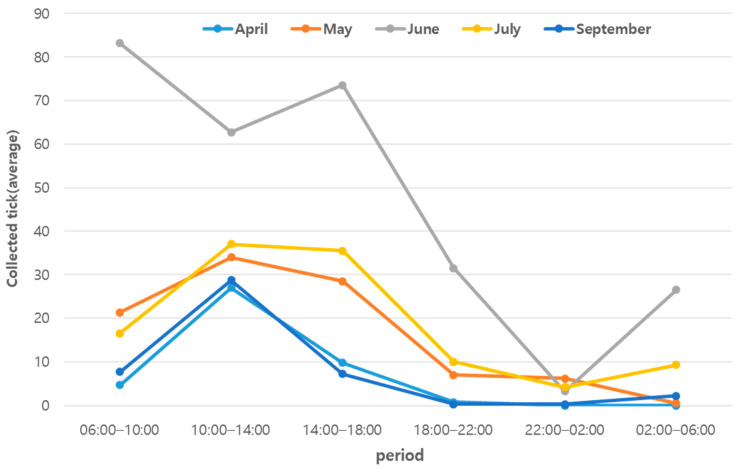
Total tick (adult + nymph) activity patterns by month (indicated by averaging the collected tick in each time period by month).

Most months showed the highest activity patterns to be between 10:00 and 14:00. In May and July, relatively high activity patterns were observed from 14:00 to 18:00. In June, tick activity had a very different pattern from that at other times, with the highest activity between 06:00 and 10:00, followed by 14:00–18:00, 10:00–14:00, 18:00–22:00, 02:00–06:00, and 22:00–02:00 (Figure 3).

**Figure 4:**
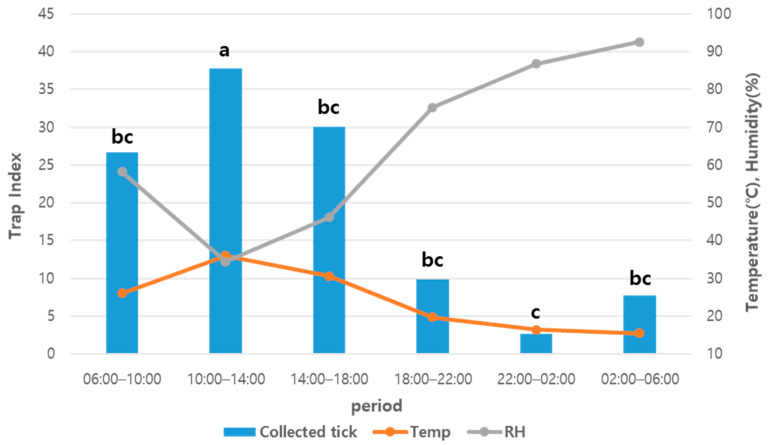
Tick activity patterns, temperature, and relative humidity by time period. Trap indexes with the same letter are not significantly different at the 5% level of probability (Duncan’s multiple range test). RH = relative humidity.

Adults and nymphs showed the highest activity between 10:00 and 14:00. At this time, the average temperature was 36.0 °C and the average relative humidity was 34.4% (Figure 4). The higher the temperature, the greater the tick activity. Tick activity was the lowest between 02:00 and 06:00, except for in June. The tick activity during this period was statistically significant compared to April, May, and September.

The activity patterns of ticks highly correlated with temperature (r = 0.679, *p* < 0.05), light intensity (r = 0.572, *p* < 0.05), and relative humidity (r = −0.361, *p* < 0.05) (Table 3).

## 4. Discussion

In the ROK, the peak developmental stage is summer for adults, spring for nymphs, and fall for larvae [52,54]. This study obtained consistent results, showing that adults were most frequently confirmed from June to July and nymphs from May to June. Additionally, 96.1% (396/412) of the identified ticks were *H. logicornis*, and a small amount of *H. flava* was collected only in September. These results are consistent with those of the other reports stating that the dominant species was *H. longicornis* [55,56].

*H*. *longicornis* is present in ten countries of the world, feeds on a variety of animal hosts, and is related to 30 human pathogens, including various species of *Rickettsia*, *Anaplasma*, *Borrelia*, *Babesia*, and viruses [17,29,34]. The prediction model revealed that *H. longicornis* could potentially spread to more extensive regions, which is important for public health and veterinary care. Therefore, ecological research on *H. longiconis* in field environments is important [57]. In this study, we confirmed the activity patterns of *H. longicornis* in the field.

The dial activity patterns of *Ixodes ricinus*, *I. scapularis*, *I. persulcatus*, and *Amblyomma americanum* were reported, which are vectors for Lyme disease [50,51,58]. Additionally, *Dermacentor variablis* (which is a vector for Rocky Mountain spotted fever and tularemia) has been studied [49]. In this study, *H. longicornis* in the ROK showed the highest activity between 10:00 and 14:00, which is similar to the activity patterns of *I. persulcatus* [58]. These different diel activity times are influenced by species, environmental factors, the climate of the region, and microhabitats [59]. 

Our study shows that *H. longicornis* preferred temperature ranges between 18 and 35 °C, and the survivable temperature is known to be 12–40 °C [16,19]. If temperatures exceed 40 °C, heat stress can cause death. When the temperature falls below 12 °C, host-seeking activities are slowed down or the ticks do not move [16]. In this study, the temperature of the research period was investigated in a wider range, between 5.7 and 42.1 °C. The temperature between 10:00 and 14:00 (the most active period) was mainly 35–40 °C. This result is consistent with *A. americanum*, *A. maculatum*, *D. andersoni*, *D. variablis*, *I. scapularis*, and *Rhipicephalus sangineus*, which have activity patterns at temperatures higher than their preferred temperatures [60]. A low activity was seen between 10:00 and 14:00 in June, because of the temperature exceeding 40 °C. Similarly, a low activity pattern appeared at temperatures below 12 °C during the nighttime in April and May. However, high activity was confirmed in June and July at 02:00–06:00, when the temperatures were approximately 20 °C.

Relative humidity is an important microclimatic factor for the survival and activity of *H. longicornis*, *A. americanum*, *I. scapularis*, *I. ricinus*, and *D. reticulatus* [49,50,51,61,62,63]. However, there was a weak correlation between relative humidity and the number of ticks collected in this study. The critical equilibrium of relative humidity for *H. longicornis* is approximately 85% [57]. When the humidity falls below this threshold, the tick continuously loses water [64], which has a negative effect on survival [65]. In this study, the humidity at the time of the highest activity was approximately 50%, and it was judged that they showed considerable activity in replenishing soil moisture before the moisture evaporated. Therefore, it seems that high relative humidity did not have a significant effect on activity patterns. Additionally, water droplets formed on the collection trap because the humidity was close to 100% at night and dawn. Occasionally, the tick was locked in water droplets. It is thought that tick activity is inhibited even when it rains or humidity is very high.

Light intensity and temperature (R = 0.802) were highly correlated. However, light intensity and activity patterns showed a low correlation (R = 0.572). Climatic factors related to light are more strongly associated with diapause than with activity. In particular, the photoperiod has a greater influence than light intensity on activity [16,66]. A long-day photoperiod triggers host-seeking behavior in *H. longicornis* in the laboratory [66]. In this study, the overall tick activity pattern was the highest in June, when the day length was the longest.

Climatic stress decreases tick survival, but increases the rate of host-seeking activity [67]. A variety of local field conditions can affect questing activity, including temperature and relative humidity [46,50], precipitation [68], time of day [69], seasonality [70,71], solar radiation [72], forest structure [73], and understory structure and invasive shrubs [74]. Additionally, several intrinsic factors (such as geographic origin and local adaptation) may influence tick-questing activity [75,76], and infected pathogens boost tick mobility [77]. For example, it is necessary to study the ecology of ticks through the analysis of various aspects as well as climatic factors. 

Also, host preferences influence tick distribution, and the survival of tick populations depends on the presence of suitable hosts [78]. *H. longicornis* is known to prefer large mammals (e.g., cattle, sheep, deer) over small ones [16]. In ROK, *H. longicornis* was identified in medium- and large-sized mammals such as cattle, goats, wild boars, roe deer, raccoons, badgers, and migratory birds [79,80,81], but not in small mammals (rodents) [82]. However, in the United State, *H. longicornis* have been found on medium- and large-sized mammals (coyotes, rabbits, raccoons, and deer), as well as small mammals (possums and marmots) [83]. Tick distribution can be influenced by several factors, including local host populations and species [84]. Although this study provided general guidelines for the highest activity of *H. longicornis*, climate changes may alter the activity of the species. The study is limited to a particular region. It is unknown if other environmental, climatic conditions and the effects of the host have a definite effect on their activity.

Ticks are associated with the transmission of various pathogens of medical and veterinary importance. They are considered one of the most important arthropod vectors after mosquitoes [85]. Our study determined the period of major daily activity of *H. longicornis*. These results will help inform the public about the risk of exposure to ticks during daytime outdoor activities. It also provides basic information for the development of tick-borne disease vector control programs.

## Figures and Tables

**Table 1:** Number of *Haemaphysalis longicornis* sampled (mean ± S.D) during the daytime and nighttime at 5 months.

	April	May	June	July	September	Total
Adults						
Daytime	6.7 ± 23.3	2.4 ± 4.0	14.3 ± 20.5	14.1 ± 8.7	2.4 ± 2.8	8.0 ± 15.2
Nighttime	0.1 ± 0.3	0.9 ± 1.7	5.6 ± 6.0	3.5 ± 3.2	0.4 ± 0.7	2.1 ± 3.4
Nymphs						
Daytime	7.2 ± 5.8	25.5 ± 39.7	58.8 ± 88.5	15.6 ± 20.9	12.1 ± 10.5	23.8 ± 47.5
Nighttime	0.2 ± 0.4	3.7 ± 6.2	14.8 ± 23.1	4.3 ± 5.8	0.5 ± 0.9	4.7 ± 12.0
Total						
Daytime	13.8 ± 25.9	27.9 ± 43.1	73.1 ± 105.9	29.7 ± 24.6	14.5 ± 13.0	31.8 ± 57.0
Nighttime	0.3 ± 0.6	4.6 ± 7.5	20.4 ± 28.1	7.8 ± 8.3	0.9 ± 1.3	6.9 ± 15.1

Student’s *t*-test for daytime and nighttime: *p* < 0.01; daytime = 06:00–18:00; nighttime = 18:00–06:00.

**Table 2:** Mean ± S.D. of the total tick (adult + nymph) and climate factors (temperature and relative humidity range).

Date	Time Period						Temperature (Mean)	Relative Humidity (Mean)
	06:00–10:00	10:00–14:00	14:00–18:00	18:00–22:00	22:00–02:00	02:00–06:00		
27 April	4.7 ± 5.1	27.0 ± 43.4	9.8 ± 7.0	0.8 ± 0.8	0.0 ± 0.0	0.0 ± 0.0	5.7–36.5 (17.8 °C)	11.0–100.0 (61.5%)
18 May	21.3 ± 39.1	34.0 ± 61.0	28.5 ± 31.4	7.0 ± 6.5	6.2 ± 10.9	0.5 ± 0.5	9.5–34.1 (20.7 °C)	16.3–85.4 (46.9%)
20 June	83.2 ± 93.0	62.7 ± 140.8	73.5 ± 96.9	31.5 ± 28.4	3.2 ± 4.0	26.5 ± 36.1	17.7–42.1 (28.4 °C)	28.3–100.0 (68.0%)
28 July	16.5 ± 8.1	37.0 ± 26.5	35.5 ± 31.3	10.0 ± 11.7	4.2 ± 2.3	9.3 ± 8.2	21.3–40.3 (28.2 °C)	39.5–100.0 (84.1%)
14 September	7.7 ± 10.3	28.7 ± 9.9	7.2 ± 3.3	0.3 ± 0.5	0.3 ± 0.5	2.2 ± 1.7	19.0–36.1 (25.2 °C)	37.2–88.0 (67.5%)
Total Mean	26.7 ± 51.5	37.8 ± 68.5	30.9 ± 50.6	9.9 ± 17.4	2.8 ± 5.5	7.7 ± 18.5	5.7–42.1 (24.0 °C)	11.0–100.0 (65.6%)

**Table 3:** Pearson’s correlation coefficients comparing three climate factors and the collected ticks.

	TEMP	HUMD	LIT
Temperature (TEMP)			
Relative humidity (HUMD)	−0.593 *		
Light intensity (LIT)	0.802 *	−0.649 *	
Collected total tick (CTT)	0.679 *	−0.361 *	0.572 *

Coefficients followed by an asterisk (*) are significant (*p* < 0.05).

## Data Availability

The original contributions presented in the study are included in the article; further inquiries can be directed to the corresponding author.
